# Broadly Neutralizing Antibodies Display Potential for Prevention of HIV-1 Infection of Mucosal Tissue Superior to That of Nonneutralizing Antibodies

**DOI:** 10.1128/JVI.01762-16

**Published:** 2016-12-16

**Authors:** Hannah M. Cheeseman, Natalia J. Olejniczak, Paul M. Rogers, Abbey B. Evans, Deborah F. L. King, Paul Ziprin, Hua-Xin Liao, Barton F. Haynes, Robin J. Shattock

**Affiliations:** a Imperial College London, Department of Medicine, Section of Virology, Group of Mucosal Infection and Immunity, London, United Kingdom; b Imperial College London, Department of Surgery, St. Mary's Hospital, London, United Kingdom; c Duke Human Vaccine Institute, Department of Medicine, and Department of Immunology, Duke University Medical Center, Durham, North Carolina, USA; Emory University

**Keywords:** HIV vaccines, human immunodeficiency virus, mucosal immunity, neutralizing antibodies, nonneutralizing antibodies

## Abstract

Definition of the key parameters mediating effective antibody blocking of HIV-1 acquisition within mucosal tissue may prove critical to effective vaccine development and the prophylactic use of monoclonal antibodies. Although direct antibody-mediated neutralization is highly effective against cell-free virus, antibodies targeting different sites of envelope vulnerability may display differential activity against mucosal infection. Nonneutralizing antibodies (nnAbs) may also impact mucosal transmission events through Fc-gamma receptor (FcγR)-mediated inhibition. In this study, a panel of broadly neutralizing antibodies (bnAbs) and nnAbs, including those associated with protection in the RV144 vaccine trial, were screened for the ability to block HIV-1 acquisition and replication across a range of cellular and mucosal tissue models. Neutralization potency, as determined by the TZM-bl infection assay, did not fully predict activity in mucosal tissue. CD4-binding site (CD4bs)-specific bnAbs, in particular VRC01, were consistent in blocking HIV-1 infection across all cellular and tissue models. Membrane-proximal external region (MPER) (2F5) and outer domain glycan (2G12) bnAbs were also efficient in preventing infection of mucosal tissues, while the protective efficacy of bnAbs targeting V1-V2 glycans (PG9 and PG16) was more variable. In contrast, nnAbs alone and in combinations, while active in a range of cellular assays, were poorly protective against HIV-1 infection of mucosal tissues. These data suggest that tissue resident effector cell numbers and low FcγR expression may limit the potential of nnAbs to prevent establishment of the initial foci of infection. The solid protection provided by specific bnAbs clearly demonstrates their superior potential over that of nonneutralizing antibodies for preventing HIV-1 infection at the mucosal portals of infection.

**IMPORTANCE** Key parameters mediating effective antibody blocking of HIV-1 acquisition within mucosal tissue have not been defined. While bnAbs are highly effective against cell-free virus, they are not induced by current vaccine candidates. However, nnAbs, readily induced by vaccines, can trigger antibody-dependent cellular effector functions, through engagement of their Fc-gamma receptors. Fc-mediated antiviral activity has been implicated as a secondary correlate of decreased HIV-1 risk in the RV144 vaccine efficacy trial, suggesting that protection might be mediated in the absence of classical neutralization. To aid vaccine design and selection of antibodies for use in passive protection strategies, we assessed a range of bnAbs and nnAbs for their potential to block *ex vivo* challenge of mucosal tissues. Our data clearly indicate the superior efficacy of neutralizing antibodies in preventing mucosal acquisition of infection. These results underscore the importance of maintaining the central focus of HIV-1 vaccine research on the induction of potently neutralizing antibodies.

## INTRODUCTION

The induction of broadly neutralizing antibodies (bnAbs) remains a key focus of human immunodeficiency virus type 1 (HIV-1) vaccine research; however, this goal has yet to be realized. Classical neutralization is thought to require binding of the antibody to the trimeric envelope spike, blocking key epitopes on the surface of the virus, inhibiting engagement with cell receptors, and preventing conformational change required for viral fusion and entry (1). During the natural course of HIV-1 infection, ∼50% of HIV-1-infected individuals develop neutralizing antibodies (nAbs) capable of inhibiting more than 50% of viral isolates (2), with ∼10% developing high levels of bnAbs capable of inhibiting 90% of viruses. While both bnAbs and nAbs require a period of months to years to develop (3), nonneutralizing antibodies (nnAbs) are found in all HIV-1-infected individuals from the acute stage of infection onwards (4). These nnAbs are thought to target a diversity of envelope structures in addition to functional trimers that include noncleaved trimers, dimers, and monomers, as well as gp41 stumps that have shed gp120. The extent to which these structures are expressed on infectious virus and/or infected cells may prove critical to any potential antiviral activity (5, 6). Nevertheless, binding of the Fc region of immunoglobulin G (IgG) to Fc-gamma receptors (FcγRs) can engage a range of effector cells capable of mediating potent antiviral activity. The extent to which Fc-effector functions can impact on initial events determining mucosal infection may prove critical to vaccine design.

Two key observations are cited in support of the potential role for Fc-effector functions contributing to mucosal protection. The first was the observation that the solid passive protection mediated by the b12 nAb against vaginal simian-human immunodeficiency virus (SHIV) challenge in the nonhuman primate model (NHP) was reduced by introducing the LALA mutation that impaired its binding to FcγRs (7). These data suggest that binding to FcγRs may augment, but is not essential for, the protective activity of nAbs. The extent to which FcγR-mediated engagement of mucosal effector cells versus the role of FcγR extending the half-life of CD4-binding site (CD4bs) antibodies contributed to the protective efficacy of b12 has yet to be resolved (8). The second key observation was that the marginal protection (31%) provided by the RV144 Thai phase III trial correlated with high concentrations of anti-V1-V2 nnAbs and an absence of nAbs against circulating viral strains (9). Although mechanistic correlates remain elusive, reduced risk correlated to Fc-mediated effector functions of nnAbs targeting the V1-V2 region of the HIV-1 envelope.

The Fc-mediated effector functions of both nabs and nnAbs are dependent upon the engagement of Fc receptors leading to activation of effector cells and further downstream activities, such as antibody-dependent cellular cytotoxicity (ADCC), antibody-dependent cellular viral inhibition (ADCVI), or antibody-dependent cellular phagocytosis (ADCP). ADCC requires engagement of FcγRs by effector cells (natural killer [NK] cells, neutrophils, and macrophages) capable of eliminating infected cells following their recognition by binding antibodies. Importantly, ADCC targets infected cells, cells that have bound virus, and cells binding shed viral envelope (5, 10, 11). The inverse correlation with infection risk and high levels of serum V1-V2 loop antibodies capable of mediating ADCC (12–14) has driven speculation that protection in RV144 was partially due to ADCC-mediating antibodies (15). ADCVI and ADCC activities likely overlap; however, ADCVI measures the additional contribution of noncytolytic mechanisms, such as FcγR-triggered production of β-chemokines that can also contribute to viral inhibition of cell-free virus (16). ADCP targets cell-free virus and prevents infection of antigen-presenting cells (macrophages and dendritic cells [DC]) through FcγR-dependent phagocytosis of opsonized viral particles (17). This may have particular relevance given the potential role of mucosal antigen-presenting cells in the uptake and subsequent presentation of HIV-1 to CD4^+^ target cells (18–21), facilitating dissemination through *cis*- and *trans*-infection pathways (22). Additionally, phagocytosis of opsonized virions may itself reduce the probability of successful infection of tissue resident CD4^+^ T cells, the primary targets of mucosal infection in humans and macaques, by reducing the half-life of infectious virus (23–25).

To date, little is known about the critical parameters mediating effective antibody blocking of HIV-1 acquisition within mucosal tissue. To bridge this gap, we assessed the relative antiviral potencies of a panel of neutralizing and nonneutralizing antibodies, including those associated with protection in the RV144 vaccine trial, across a range of tissue and cellular models designed to mimic the initial events required to establish mucosal infection. To assess the relative efficacy of bnAbs, we chose representative monoclonal antibodies (MAbs) targeting four major sites of envelope vulnerability: the CD4-binding site (b12, VRC01, and CH31), membrane-proximal external regions (MPERs) (2F5 and 4E10), V1-V2 glycan Env regions (PG9 and PG16), and outer domain glycans (2G12). To assess the potential of nnAbs to block infection and/or onward transmission from mucosal tissue, we selected two individual nnAbs targeting the C1 region of gp120 (A32) and cluster I of gp41 (4B3) previously reported to show high levels of ADCC and ADCP in *in vitro* assays (26, 27). In addition, we assembled three nnAb combinations. Combination 1 was 7B2/CH58/CH90, targeting the principal immunodominant domain (PID) of gp41 (7B2), the V2 region of gp120 (CH58), and the CD4-induced (CD4i) cluster 1 region (CH90); all are known to display ADCC activity in a range of *in vitro* models (15, 28), and 7B2 in combination with CH58 shows enhanced capacity to capture of infectious virions (29). Combination 2 was 7B2/CH58/CH22, combining 7B2 and CH58 with CH22 targeting the V3 region of gp120, also with known ADCC activity and limited tier 1 neutralization (30). Combination 3 was F240/M785-U1/N10-U1, all focused on different epitopes within the C1 region of gp41 and previously shown to exhibit ADCC activity (31, 32).

## RESULTS

### TZM-bl and peripheral blood mononuclear cell (PBMC) assays differentiate FcR-dependent function.

**FIG 1:**
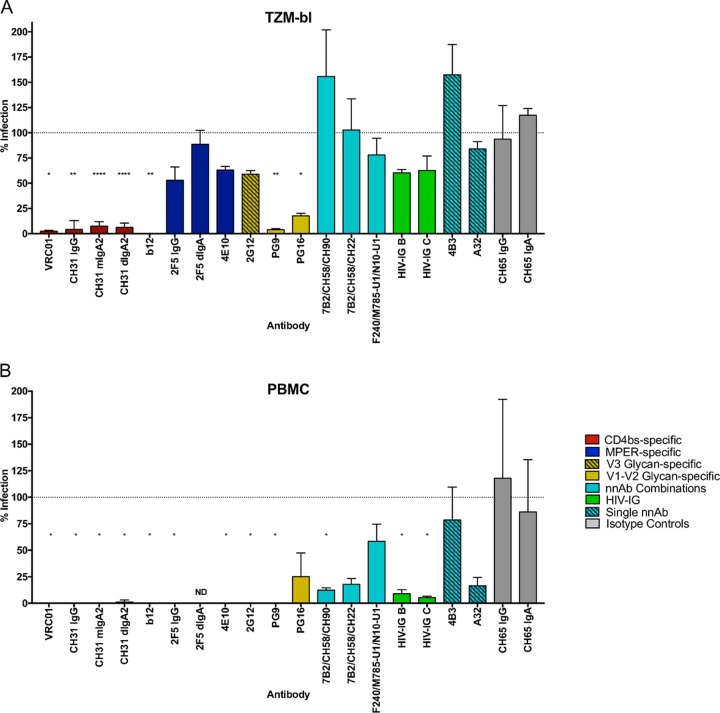
Inhibition of single antibodies and antibody combinations in TZM-bl cells and PBMC. Shown are results for inhibition of HIV-1_BaL_ by antibody panels (50 μg/ml of single antibodies; 25 μg/ml of each in combinations) in the direct infection of TZM-bl (*n* = 3) (A) and PBMC (*n* = 3) (B). Data are presented as percent infection compared to the HIV-1_BaL_-positive control. One-way ANOVA with Dunnett's multiple-comparison test followed by an unpaired *t* test was used to compare the antibodies with the CH65 isotype controls. ND, not done. *, *P* < 0.05; **, *P* < 0.01; ***, *P* < 0.001; ****, *P* < 0.0001.

Initial studies assessed the ability of antibodies to block HIV-1_BaL_ infection using an indicator cell line (TZM-bl) devoid of FcR. Known bnAbs VRC01, CH31, b12, PG9, and PG16 demonstrated significant reduction in infection (Fig. 1A and Table 1). The inhibitory activity of CH31 was reduced when presented as monomeric IgA2 (mIgA2) or dimeric IgA2 (dIgA2) compared to IgG (Table 1). In contrast, MPER bnAbs failed to demonstrate significant inhibition in the absence of FcR engagement, while 2G12 provided only a modest reduction in infection at the highest concentration tested (50 μg/ml). None of the nnAbs or HIV-IG preparations demonstrated inhibition in the absence of FcR.

**TABLE 1:**
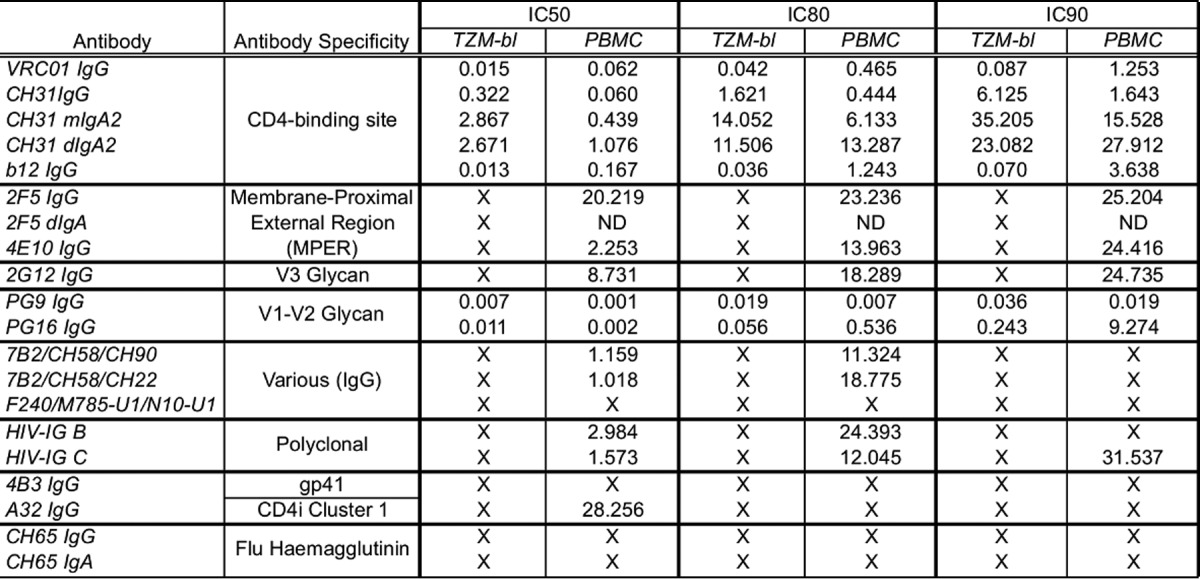
Summary of HIV-1_BaL_ neutralization data in TZM-bl cells and PBMC^*a*^

a IC_50_s, IC_80_s, and IC_90_s (in micrograms per milliliter) were determined from neutralization data of HIV-1-specific antibodies in TZM-bl cells (*n* = 3) or PBMC (*n* = 3). X, no neutralization seen at 25 μg/ml (antibody combinations) or 50 μg/ml (single antibodies). ND, not done.

bnAbs active in the TZM-bl assay were also active in PBMC (Fig. 1B) and, although individual antibodies showed some differences in potency between the two assays (Table 1), there was no evidence that the presence of FcR in the PBMC assay had a major impact on activity. In contrast, the activity of MPER bnAbs 2F5 IgG and 4E10 and glycan-specific 2G12 showed enhanced activity in PBMC cultures. Strikingly, the nnAb combinations, nnAb A32, and both HIV-IG B and C pools demonstrated measurable levels of HIV-1_BaL_ inhibition in PBMC cultures (Fig. 1B and Table 1), although only HIV-IG C generated a 90% inhibitory concentration (IC_90_).

### Antibody inhibition in macrophage and dendritic cell cultures.

**FIG 2:**
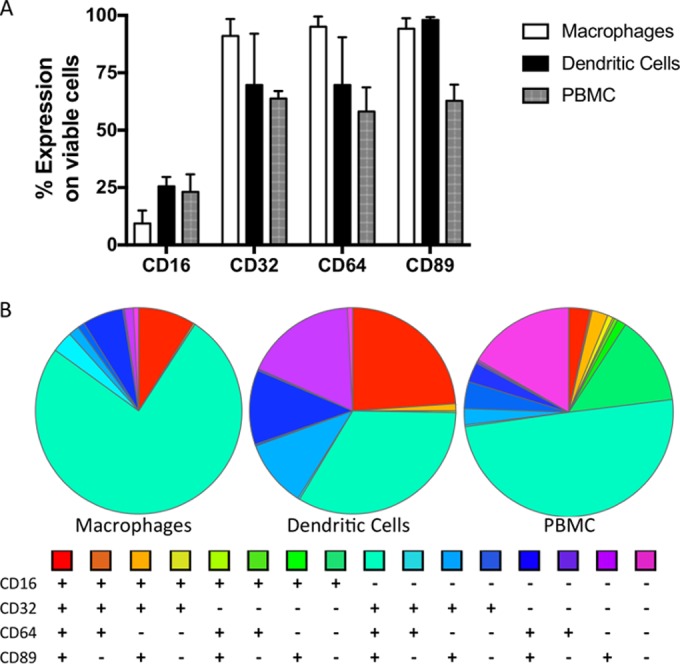
Fc receptor phenotyping of macrophages, dendritic cells, and PBMC used in the cellular inhibition assays. (A) Flow cytometry analysis of the percent expression of CD16, CD32, CD64, and CD89 FcR expression on total viable macrophages, dendritic cells, and PBMC used in the cellular inhibition assays (*n* = 3). (B) Boolean gating analysis of the FcR expression on total viable cells to show the combinatorial variability of the Fc receptors.

**FIG 3:**
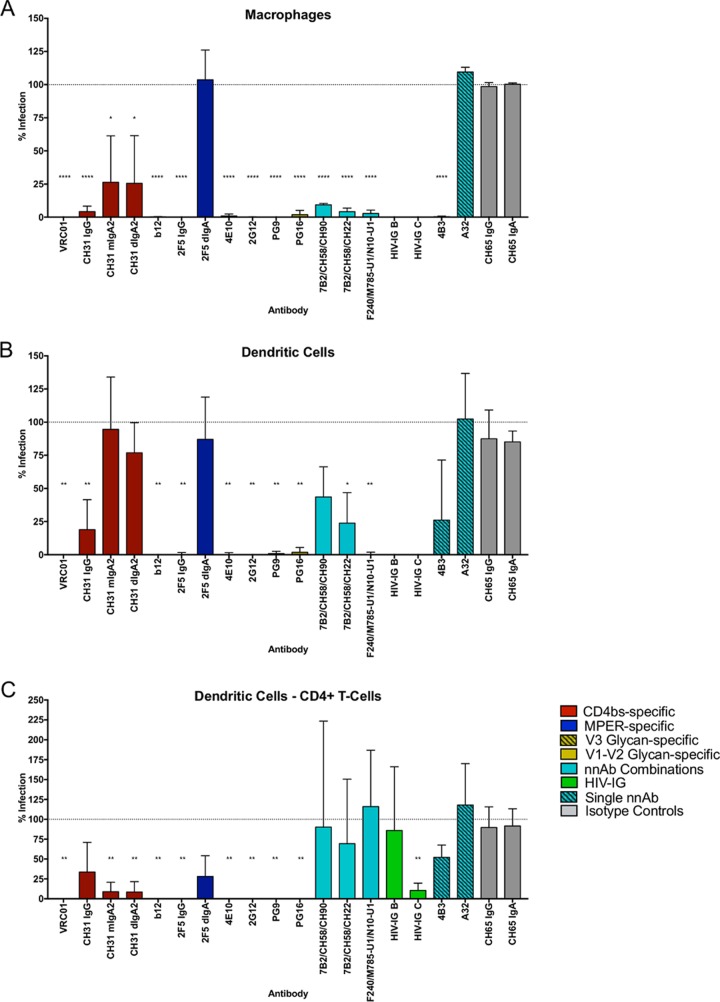
Inhibition of single antibodies and antibody combinations in macrophages, dendritic cells, and DC-to-CD4 T-cell inhibition assays. Shown are results of inhibition of HIV-1_BaL_ by antibody panels (50 μg/ml of single antibodies; 25 μg/ml of each in combinations) in the direct infection of macrophages (*n* = 3) (A) and dendritic cells (*n* = 3) (B) and *trans*-infection of CD4^+^ T cells from dendritic cells (*n* = 3) (C). (For HIV-IG B and C, *n* = 1 in panels A and B.) Data are presented as percent infection compared to the HIV-1_BaL_-positive control. One-way ANOVA with Dunnett's multiple-comparison test followed by an unpaired *t* test was used to compare the antibodies with the CH65 isotype controls. *, *P* < 0.05; **, *P* < 0.01; ***, *P* < 0.001; ****, *P* < 0.0001.

To further investigate the ability of the antibodies to block HIV-1_BaL_ infection in FcR-positive cells, subsequent experiments were performed in macrophage and dendritic cell models, as previously described (24). Fc receptor expression was determined for the different cellular models (Fig. 2). All IgG bnAbs displayed potent inhibition of macrophage infection (Fig. 3A). Interestingly, IgA forms of CH31 were less effective than IgG, while 2F5 dIgA was completely inactive. With the exception of A32, all nnAbs and both HIV-IG preparations were effective against macrophage infection. A similar pattern was evident for direct infection of dendritic cells. However, the difference in activity between CH31 IgG and both mIgA2 and dIgA2 was more pronounced, while the activity of some of the nnAbs failed to reach significance (4B3 and 7B2/CH58/CH90) (Fig. 3B). To complement these studies, the ability of antibodies to inhibit *trans*-infection from DC to CD4^+^ T cells was also assessed. All IgG bnAbs were effective against *trans*-infection. IgA versions were also active in this assay (Fig. 3C), reflective of their activity in TZM-bl cells (Fig. 1A). In contrast, nnAbs and HIV-IG B failed to show significant inhibition, the exception being HIV-IG C, which reduced *trans*-infection by 89.5% (standard deviation [SD], 9.2; *P* < 0.01 [Table 2]).

**TABLE 2:**
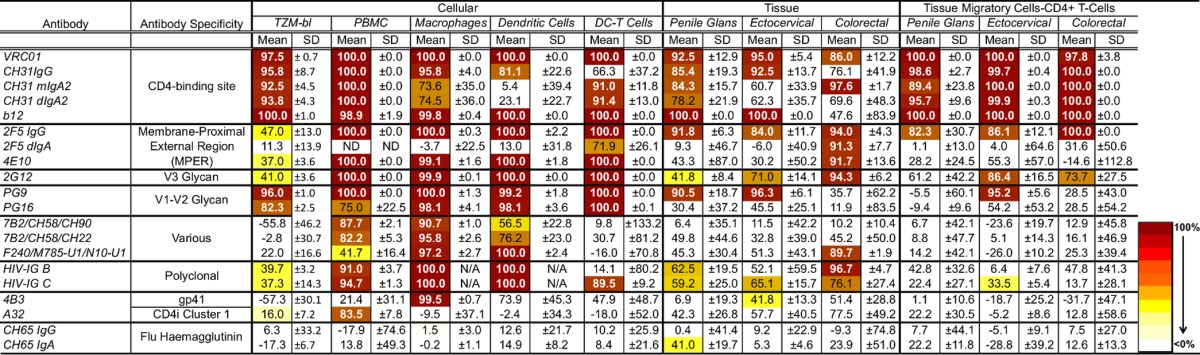
Summary of percentage HIV-1_BaL_ inhibition for all inhibition assays^*a*^

a Percent inhibition of single antibodies (50 μg/ml) and antibody combinations (25 μg/ml each). Means and SDs are included. Highlighted cells indicate inhibition of >2 SD. ND, not done; N/A, not available (*n* = 1).

### Inhibitory activity in mucosal tissue explants.

**FIG 4:**
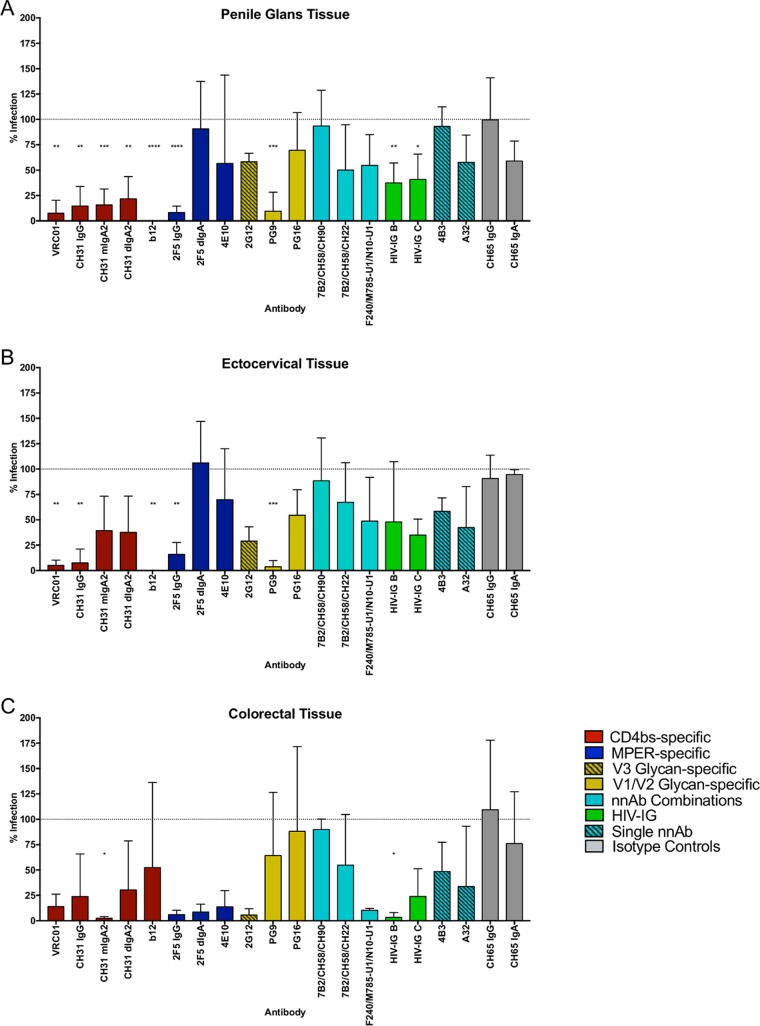
Inhibition by single antibodies and antibody combinations in mucosal tissue explants. Shown are results of inhibition of HIV-1_BaL_ by antibody panels (50 μg/ml of single antibodies; 25 μg/ml of each in combinations) in the direct infection of penile glans (*n* = 3) (A), ectocervical (*n* = 2 for HIV-IG B and C; *n* = 3 for remaining Abs) (B), and colorectal (*n* = 3) (C) tissues. Data are presented as percent infection compared to the HIV-1_BaL_-positive control. One-way ANOVA with Dunnett's multiple-comparison test followed by an unpaired *t* test was used to compare the antibodies with the CH65 isotype controls. *, *P* < 0.05; **, *P* < 0.01; ***, *P* < 0.001; ****, *P* < 0.0001.

To model the activity of antibodies at the mucosal portals of infection, their potential to inhibit direct HIV-1_BaL_ infection of mucosal tissue explant cultures (penile glans, ectocervical, and colorectal) was assessed (24). The location, phenotype, and number of cells expressing the range of FcR and their relative levels of expression across these three different tissue models are described in reference 33. All CD4bs bnAbs were active across the three explant models (Fig. 4), with statistical significance in penile and cervical tissues. The lack of significance in colorectal tissue, despite major reductions in HIV-1_BaL_ infection, reflects the high variability in levels of infection in the positive controls (Fig. 4 and Table 2). MPER-specific 2F5 IgG also displayed potent inhibition across the different tissue models, as previously described (34); however, 2F5 dIgA failed to show activity in the penile and cervical models. MPER-specific 4E10 appeared to be active only in colorectal tissue (Fig. 4C). The activity of glycan-specific bnAbs was more mixed. PG9 demonstrated good activity in penile and cervical tissues but poor activity in colorectal explants. In contrast, 2G12 was active only in colorectal tissue, while PG16 was ineffective at preventing infection in any of the tissue models. nnAbs failed to demonstrate significant inhibition in any of the mucosal tissue models (Fig. 4). However, a major reduction of infection was observed for the F240/M785-U1/N10-U1 combination (89.7% ± 1.9% [Table 2]). HIV-IG B and C displayed a range of reductions in infection across the models, reaching significance in cervical (B and C) and colorectal (B only) tissues.

### Inhibition of *trans*-infection by mucosal tissue emigrants.

**FIG 5:**
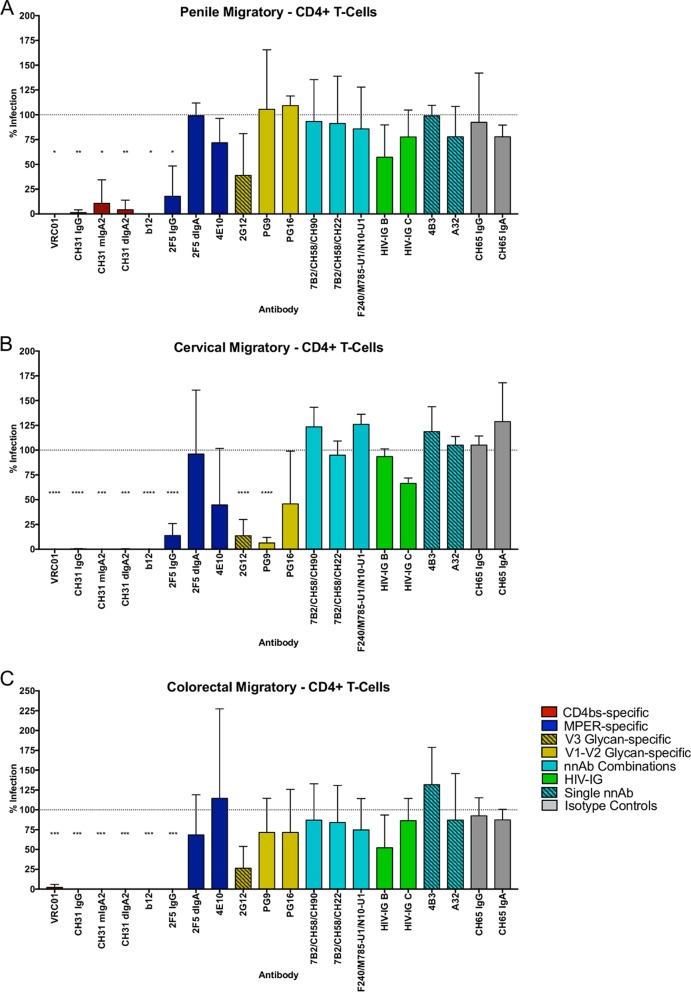
Inhibition by single antibodies and antibody combinations of HIV-1_BaL_ infection from mucosal migratory cells to CD4^+^ target cells. Shown are results of inhibition of HIV-1_BaL_ by antibody panels (50 μg/ml of single antibodies; 25 μg/ml of each in combinations) in preventing the onward infection of CD4^+^ T cells by migratory cells isolated from penile glans (*n* = 3) (A), ectocervical (*n* = 2 for HIV-IG B and C; *n* = 3 for remaining Abs) (B) and colorectal (*n* = 3) (C) tissues. Data are presented as percent infection compared to the HIV-1_BaL_-positive control. One-way ANOVA with Dunnett's multiple-comparison test followed by an unpaired *t* test was used to compare the antibodies with the CH65 isotype controls. *, *P* < 0.05; **, *P* < 0.01; ***, *P* < 0.001; ****, *P* < 0.0001.

A critical step in HIV-1 transmission may be the dissemination of infection beyond the initial foci of primary infection. Using an established model designed to mimic these events (24), cells migrating out of tissue explants following HIV-1_BaL_ viral exposure were collected and cocultured with CD4^+^ target cells in the presence of antibody (Fig. 5). Across all tissue models, CD4bs-specific antibodies were very efficient at blocking onward transmission of HIV-1_BaL_ to the CD4^+^ target cells, with levels of inhibition ranging between 89.4 and 100% for IgG and IgA forms. The MPER-specific antibodies were less efficient at inhibiting infection, with only 2F5 IgG demonstrating any significant ability to block onward infection of HIV-1_BaL_ across the three models. Glycan-specific antibodies showed a more diverse ability to inhibit onward infection, where significant reduction was observed only for 2G12 and PG9. nnAbs and HIV-IG B and C failed to inhibit onward infection in any of the migratory tissue cell models (Fig. 5).

## DISCUSSION

This study set out to investigate the functional activity of neutralizing and nonneutralizing antibodies in a series of cellular and tissue explant models designed to mimic the early transmission events at the mucosal portals of infection.

CD4bs-specific bnAbs were efficient at inhibiting HIV-1_BaL_ infection across all models. There was little difference in activity between PBMC and TZM-bl assays, suggesting that inhibitory activity was not dependent upon of FcγR engagement. Monomeric and dimeric IgA versions of CH31, unable to engage FcγR, were also active across the models. Furthermore, all CD4bs bnAbs were active against cell-cell transmission in both cellular and tissue models. The observation that VRC01 provided the most consistent inhibition across all infection models underscores the potential importance of the conserved CD4bs as a target for vaccine design (35) and monoclonal prevention strategies (36). These data mirror passive-infusion experiments with nonhuman primates (NHPs) demonstrating potent efficacy against mucosal challenge (36).

V1-V2 glycan-specific bnAbs, PG9 and PG16, demonstrated good levels of inhibition across all cellular models but more variable results in tissue models. PG9 demonstrated good levels of inhibition in penile glans and ectocervical tissues but was less effective against direct infection of colorectal tissue and was active only against onward transmission by ectocervical migratory cells. PG16, in contrast, was ineffective against HIV-1_BaL_ infection in all tissue models. Differences across tissues may reflect potential reactivity with mammalian carbohydrates where PG16 binds complex-type glycans more tightly than Man5GlcNAc2, while PG9 prefers Man5GlcNAc2 (37); alternatively, this may reflect known differences in trimer dependence (38, 39): PG9 is able to weakly bind monomeric gp120 in addition to trimeric Env, but PG16 is able to bind only the latter. An alternative *ex vivo* study using colorectal and ectocervical tissue explants demonstrated sustained inhibition of viral replication by PG9 and PG16 in an ectocervical tissue model but loss of viral control within the colorectal tissue after 21 days in culture (40). Significant differences in methodology are likely to explain the variance in the observed levels of inhibition; nevertheless, viral rebound in colorectal tissue reflects the lack of inhibition observed in our study. Heterogeneity in glycosylation of HIV-1 Env leaves these antibodies vulnerable to viral escape (41, 42). The higher levels of HIV-1 replication in colorectal tissue likely enhance the chance of observing viral outgrowth by the proportion of virions not recognized by these glycan-dependent antibodies. The lower potency of PG9 relative to VRC01 mirrors that seen in NHP passive-infusion studies (36).

Interestingly, the V3 glycan-specific antibody 2G12, although performing poorly for classical neutralization in the TZM-bl assay, performed well across all inhibition assays, with the notable exception of penile glans tissue, and exerted similar levels of inhibition against onward transmission by migratory cells across the tissue types. Lack of activity in TZM-bl cells likely reflects the kinetics of 2G12 neutralization (43). The potency of 2G12 in mucosal tissue explants concords with that seen in NHP passive-protection studies (44).

The MPER-specific bnAb 2F5 IgG also performed well across all cellular and tissue models (Table 2), consistent with the results of *in vivo* NHP challenge experiments (34, 45), but was ineffective in the TZM-bl assay. Interestingly, 2F5 dIgA was ineffective in the majority of assays, with the notable exception of colorectal tissue. These observations confirm previous studies showing reduced activity for polymeric dIgA and pIgM isotypes (46, 47) and likely reflect the known influence of FcγR engagement on the neutralizing activity of 2F5 IgG (48). In contrast, MPER-specific 4E10, despite demonstrating good levels of inhibition across cellular models, was only able to inhibit viral infection in colorectal tissue and was inactive against onward transmission by migratory cells. The inhibitory activity of 4E10 in the colorectal tissue is in concordance with NHP studies demonstrating that 4E10 protects against intrarectal challenge with SHIV_BaL_ (45). The weak activity of 4E10 against onward dissemination of virus reflects earlier reports of poor activity against cell-to-cell spread of HIV-1 (49). The lack of activity in the cervical and penile glans tissues is in concordance with similar observations in an alternative cervical tissue model (40). The predictive nature of these observations is unclear given that 4E10, as a single monoclonal antibody, has not been tested against vaginal challenge in the SHIV model. It is also possible that potential polyreactivity for mammalian cells could have influenced activity in these different mucosal models (50).

To assess the potential of Fc-mediated effector functions to block infection and/or onward transmission from mucosal tissue, we selected two individual nnAbs (4B3 and A32) previously reported to show high levels of ADCP and ADCC in *in vitro* assays (26, 27). 4B3 displayed inhibitory activity only in macrophages, as previously described (27), while A32 was active only in PBMC cultures. Critically, neither antibody displayed potent inhibition in any of the mucosal tissue models.

The three nnAb combinations demonstrated variable levels of inhibition in PBMC cultures, good levels of inhibition in macrophage cultures, and again more variable levels in dendritic cell models. These antibodies were less active when assessed individually (data not shown), supporting previous observations that specific antibody combinations synergize for increased antiviral activity (51). Both macrophages and dendritic cells can mediate ADCP, ADCC, and ADCVI (reviewed in reference 52). These data extend previous observations of the inhibitory potential of 7B2, CH22, and F240 in macrophage and dendritic cell models (28, 53) and likely reflect the high levels of FcγR expression on these *in vitro*-derived cells (Fig. 2). In contrast, none of the nnAb combinations were effective at preventing onward cell-cell transmission by *in vitro*-derived DC or tissue migratory cells. Furthermore, the nnAb combinations were unable to significantly inhibit infection across the three tissue models, with one notable exception in which the third combination (F240/M785-U1/N10-U1) was able to reduce infection of colorectal explants by 89.7% (±1.9%).

The trend for increased activity with increased polyclonality was also evident for the two polyclonal HIV-IG sera (B and C). While both sera displayed little activity with respect to classical neutralization in the TZM-bl assay, both demonstrated robust activity across PBMC, macrophage, and dendritic cell cultures and variable levels of inhibition in the three tissue models, providing the highest levels of inhibition in colorectal tissue. However, both were ineffective in blocking onward transmission by tissue migratory cells to CD4^+^ T cells.

These data demonstrate some important features. First, activity in the TZM-bl assay showed a moderate correlation to activity in penile and cervical tissue models (*R*^2^ = 0.64 and *P* = 0.0001 and *R*^2^ = 0.54 and *P* = 0.0007, respectively). However, there was no correlation (*P* < 0.05; not significant) with activity in the colorectal model. Furthermore, the TZM-bl assay was not fully predictive of inhibition for some specific antibodies, specifically 2F5 and 2G12 being more active and PG9 and PG16 less active in tissue versus TZM-bl cells. Second, activity of nnAbs in PBMC, macrophage, and dendritic cell assays did not translate to equivalent activity in tissue, and there was no correlation for any of these cellular models with activity in tissue. There was, however, an apparent trend for increased inhibition with nnAb polyclonality (HIV-IG > MAb combinations > individual MAbs). Nevertheless, activity of nnAbs in tissue was low to absent, with the notable exceptions of HIV-IG (B and C) and a MAb combination (F240/M785-U1/N10-U1) in colorectal tissue. It is, however, possible that these modest effects might contribute to activity of bnAbs in the context of polyclonal response to vaccination; assessment of bnAbs in combination with nnAbs merits further investigation.

ADCC, ADCP, and ADCVI have been proposed as potential mechanisms by which antibodies (both nAb and nnAbs) might impact transmission events. It is clear from the current study that individual nnAb with known ADCC activity (26), while active in PBMC cultures, were ineffective in mucosal tissues. This likely reflects the very low numbers of resident ADCC effector cells within these tissues (33). Likewise, nnAbs with known potent ADCP and ADCVI activity (26, 27) were also ineffective at protecting mucosal tissue from infection. Again, these results reflect low FcR expression in penile and cervical tissues, while the increased activities of the two HIV-IGs mirror the trend for a higher number of effector cells in colorectal tissue as reported in our recent characterization of FcR expression across these different models (33). Interestingly, the trend for increased antiviral activity in colorectal tissue was also observed for 4E10 IgG and 2F5 dIgA. These data, combined with our earlier studies (33), suggest that resident effector cell numbers and low FcγR expression limit the potential of nnAbs to prevent the initial foci of infection within these mucosal tissue sites. This is perhaps unsurprising given that the predominant targets of infection are CD4^+^ T cells that lack FcγR expression (23–25). This is compounded by the inability of nnAbs or HIV-IG to block onward cell-cell transmission by migratory cells emigrating from these tissue sites, likely essential in rapid dissemination of infection (54). These data reflect previous cellular studies that demonstrate that nnAbs are ineffective against cell-cell transmission (55). The observed inferiority of nnAbs to block initial mucosal infection is in concordance with the reported inability of nnAb and HIV-IG to prevent mucosal SHIV acquisition in NHP studies (27, 28, 56, 57), contrasting with the sterile protection mediated by a number of bnAbs (58).

A number of limitations should be considered when interpreting this study. First, experiments were performed with a single viral isolate (HIV-1_BaL_) known to replicate efficiently in primary cells (CD4 T cells, macrophages, and dendritic cells) while universally targeting CD4 T cells in mucosal models (23, 59, 60). Furthermore, HIV-1_BaL_ is a tier 1B neutralization-sensitive virus; therefore, while the observed superiority of bnAbs in preventing HIV-1 infection will certainly be generally applicable, the activity of individual monoclonal antibodies is likely to vary with different viral isolates and their neutralization sensitivities. Nevertheless, the approach described in this publication provides an important benchmark for evaluation of additional antibodies and viral strains. Second, the high variability in viral replication between tissue donors means that lower, but potentially meaningful, levels of inhibition may not be apparent in this model; further, the maximal concentration of antibody used (50 μg/ml) does not preclude potential inhibition with higher concentrations. Third, all IgG antibodies were expressed within a common recombinant IgG1 isotype. We cannot exclude that expression within a different isotype backbone, in particular IgG3, with a greater potential to engage FcR effector cells (61, 62) might have generated different results. Finally, and perhaps most importantly, the tissue models used in this study assess only the impact of resident effector cells on the establishment of mucosal infection. This precludes the potential influx of effector cells, such as neutrophils, natural killer cells, macrophages, and dendritic cells, in response to the chemotactic signals induced by HIV-1 infection (63). The potential of infiltrating FcγR effector cells to modulate the establishment of infection warrants further investigation. It is of interest to note that R. Astronomo et al. have obtained results similar to those reported here in explant cultures *in vitro* and have confirmed limited protection of nonneutralizing antibodies *in vivo* with high-dose intrarectal challenges (unpublished data). Furthermore, previous NHP studies have suggested that while nnAbs have an ability to limit the number of transmitter/founder viruses (28) and reduced the viral set point postinfection (27), they are inferior to bnAbs in preventing acquisition of infection.

This study provides important mechanistic insight on the differential activities of bnAbs and nnAbs in preventing infection at the mucosal tissue level. The solid protection provided by bnAbs, in particular those targeting the CD4bs, clearly demonstrates their superior potential over nonneutralizing antibodies for preventing HIV-1 infection at the mucosal portals of infection.

## MATERIALS AND METHODS

### Antibodies and reagents.

Antibodies b12, 4E10, 2F5 IgG and dIgA, PG9, PG16, 4B3, and 2G12 were obtained from Polymun Scientific, GmbH (Austria). Expression plasmids for antibody VRC01 were obtained from Dennis Burton, Scripps Research Institute (La Jolla, CA), and antibodies were produced as recombinant IgG1 in 293T cells. A32, 7B2, CH22, CH31, CH58, and CH90 were produced as recombinant IgG1 (also CH31 mIgA2 and dIgA2 [64]) in CHO cells, as previously described (28, 61). A32, 7B2, CH90, and CH22 contained the AAA mutations (S298A, E333A, and K334A) optimized for binding to FcγRIIIa (CD16) and to augment antibody ADCC activity (65). The CH65 isotype control is an IgG1 bnAb recognizing influenza virus hemagglutinin (66). F240, M785-U1, and N10-U1 IgG1 were kindly provided by George Lewis (Institute of Human Virology, Baltimore, MD). HIV-IG B and C were kindly provided by David Montefiori, Duke University. The following reagents were obtained through the NIH AIDS Research and Reference Reagent Program, Division of AIDS, NIAID, NIH: TZM-bl cells were from John C. Kappes, Xiaoyun Wu, and Tranzyme Inc; PM-1 cells were from Paulo Lusso and Robert Gallo; and HIV-1_BaL_ was donated by Suzanne Gartner, Mikulas Popovic, and Robert Gallo.

### Tissue samples.

Ectocervical tissue was acquired from women undergoing planned therapeutic hysterectomy at St. Mary's Hospital (London, United Kingdom). Penile glans tissue was acquired from men undergoing elective gender reassignment surgery at Charing Cross Hospital (London, United Kingdom). Surgically resected specimens of colorectal tissue were collected at St. Mary's Hospital from patients undergoing rectocele repair and colectomy for colorectal cancer. Only healthy tissue obtained 10 to 15 cm away from any tumor was employed.

### Ethics statement.

Written informed consent was obtained from all donors. All tissues were collected under protocols approved by the Imperial College NHS Trust Tissue Bank and the National Research Ethics Committee in accordance with the Human Tissue Act 2004. Approval for this project was granted by the Imperial College Healthcare Tissue Bank, under their HTA research license, and ethics thus conveyed through this process by the Multi Research Ethics Committee (MREC), Wales.

### Cell lines.

TZM-bl cells (NIH AIDS Reagent Program) were cultured in complete Dulbecco modified Eagle medium (DMEM) in a 95% humidified incubator with 5% CO_2_ at 37°C. PM-1 cells (NIH AIDS Reagent Program) were grown in suspension in complete RPMI medium in a 95% humidified incubator with 5% CO_2_ at 37°C.

### TZM-bl neutralization assay.

TZM-bl cells were utilized to assess the potential neutralizing ability of antibodies to HIV-1_BaL_. Test antibodies were titrated 1:4 from a starting concentration of 50 μg/ml. Positive control (virus plus cells only) and negative control (cells only) wells were included in the assay setup. The cells were incubated in a 95% humidified incubator with 5% CO_2_ at 37°C for 36 to 72 h.

Postincubation, all supernatant was removed from the cells, which were washed once with 200 μl of phosphate-buffered saline (PBS). Luciferase lysis buffer was diluted 1:5 with distilled water (dH_2_O), and 100 μl was added to all wells. Plates were placed at −80°C for at least 2 h to allow for full lysis. Postlysis, the plates were thawed and 50 μl of the lysate was transferred to a white 96-well high-binding plate. Luciferase substrate was reconstituted by adding 10 ml of luciferase buffer to the lyophilized substrate. Fifty microliters of luciferase substrate was added to the lysate and mixed well. Plates were read immediately in relative light units (RLU) using a FLUOstar Omega plate reader (BMG Labtech, United Kingdom). Ninety percent, 80%, and 50% inhibitory concentrations (IC_90_, IC_80_, and IC_50_, respectively) were calculated according to linear regression of the antibody titration using GraphPad Prism7.

### PBMC.

Peripheral blood mononuclear cells (PBMC) were obtained from leukocyte cones (NHS Blood and Transplant, Collingdale, United Kingdom). Leukocytes were separated by Histopaque (Sigma, United Kingdom) gradient centrifugation. Before HIV-1 infection, PBMC were activated with 5 μg/ml of phytohemagglutinin (PHA; Sigma, United Kingdom) and 10 U/ml of interleukin-2 (IL-2; Novartis, United Kingdom) in complete RPMI medium for 3 days.

### Monocyte-derived macrophages (MDM).

Freshly isolated PBMC were washed and resuspended at 3 × 10^6^/ml in serum-free AIM-V medium containing 20 ng/ml of granulocyte-macrophage colony-stimulating factor (GM-CSF). Cells were seeded at 3 × 10^5^ in 100 μl in flat-bottomed high-binding 96-well plates and incubated at 37°C (5% CO_2_). After 3 to 4 days, fresh serum-free AIM-V medium supplemented with 20 ng/ml of GM-CSF was added and the cells were cultured for a total of 7 days.

### Monocyte-derived dendritic cells (MDDC).

Freshly isolated PBMC were used to separate mononuclear cells by CD14 positive selection using an AutoMacs separation system. Twenty microliters of CD14 MicroBeads was added per 10^7^ cells and incubated for 20 min at 4°C. Cells were washed once with AutoMacs running buffer by centrifugation and resuspended in 2 ml of AutoMacs running buffer for magnetic separation. CD14-positive cells were resuspended in 40 ml of complete RPMI medium supplemented with 30 ng/ml of IL-4 and 25 ng/ml of GM-CSF and cultured for 3 to 4 days at 37°C (5% CO_2_). Medium was replaced after 3 to 4 days with fresh complete RPMI medium supplemented with 30 ng/ml of IL-4 and 25 ng/ml of GM-CSF and incubated for a further 3 days at 37°C (5% CO_2_).

### Flow cytometry staining.

MDM, MDDC, or PBMC were stained using a multicolored flow cytometry panel designed to determine Fc receptor expression. Cells were stained with CD3 V450 (UCHT1), CD14 Qdot 605 (T[u]K4) (Invitrogen), CD16 Pacific Orange (3G8) (Invitrogen), CD11c A700 (B-ly6), CD123 phycoerythrin (PE)-Cy5 (9F5), CD32 allophycocyanin (APC) (FLI8.26), CD64 APC H7 (10.1), CD89 PE (A59), and CD19 fluorescein isothiocyanate (FITC) (HIB19). Unless otherwise specified, all antibodies were sourced from BD Biosciences. Dead cells were excluded from analysis through staining with Aqua viability dye (Invitrogen).

### Flow cytometry acquisition and analysis.

Samples were acquired using an LSRIIFortessa fluorescence-activated cell sorter (FACS) (BD Biosciences) and analyzed using FlowJo (Tree Star, Ashland, OR) and PESTLE and SPICE (National Institute of Allergy and Infectious Diseases, USA). Compensation matrices were created on FlowJo using single-stained anti-mouse Ig, κ/negative-control compensation beads (BD Biosciences).

### MDM inhibition assay.

Antibodies were prepared at 100 μg/ml of AIM-V medium and incubated 1:1 with cell-free HIV-1_BaL_ (10^4^ 50% tissue culture infective doses [TCID_50_]) for 1 h at 37°C. The virus-antibody suspension was added to 7-day-old macrophage cultures and incubated for 2 h at 37°C. Postincubation, cells were washed 3 times with PBS and antibodies at 50 μg/ml in 200 μl of AIM-V medium plus 20 ng/ml of GM-CSF. The cells were further incubated for 7 days in a 95% humidified incubator with 5% CO_2_ at 37°C. All assays were performed in triplicate and included controls: medium-only, virus-only, and antibody isotype controls at the same concentration as the test antibodies.

### MDDC inhibition assay.

Antibodies were prepared at 100 μg/ml in complete RPMI medium and incubated 1:1 with cell-free HIV-1_BaL_ (10^4^ TCID_50_) for 1 h at 37°C. A total of 4 × 10^4^ MDDC were added in 100 μl of complete RPMI medium to the virus-antibody suspension and incubated overnight 37°C. Postincubation, cells were washed 3 times with PBS by centrifugation and antibodies were added at 50 μg/ml in 200 μl of complete RPMI medium plus 30 ng/ml of IL-4 and 25 ng/ml of GM-CSF. The cells were further incubated for 7 days in a 95% humidified incubator with 5% CO_2_ at 37°C. All assays were performed in triplicate and included controls: medium-only, virus-only, and antibody isotype controls at the same concentration as the test antibodies.

### MDDC–PM-1 T cell *trans*-infection inhibition assay.

MDDC at 2 × 10^4^ per well were incubated with cell-free HIV-1_BaL_ (10^4^ TCID_50_) for 1 h at 37°C. Postincubation, MDDC were washed 3 times with PBS by centrifugation and 50 μg/ml of antibody was incubated with the cells for 30 min at 37°C. Postincubation, 4 × 10^4^ PM-1 T cells were added per well and the cells were further incubated for 7 days in a 95% humidified incubator with 5% CO_2_ at 37°C. All assays were performed in triplicate and included controls: medium-only, virus-only, and antibody isotype controls at the same concentration as the test antibodies.

### Mucosal tissue explant inhibition assay.

Antibodies were prepared at 100 μg/ml of complete RPMI medium and incubated 1:1 with cell free HIV-1_BaL_ (2 × 10^4^ TCID_50_) for 1 h at 37°C. Tissue explants were cut to 3 mm^3^ and added to the virus/antibody suspension for overnight incubation at 37°C. Postincubation, tissues were washed 3 times with PBS and antibodies were added at 50 μg/ml in 200 μl of complete RPMI medium. The tissues were further incubated for a total of 21 days in a 95% humidified incubator with 5% CO_2_ at 37°C, with feeding every 3 to 4 days. All assays were performed in triplicate and included controls: medium-only and antibody isotype controls at the same concentration as the test antibodies.

### Mucosal migratory cells.

Post-overnight infection, migratory cells were collected from the tissue explants and washed three times with PBS by centrifugation (67, 68). The cells were further incubated with 50 μg/ml of antibody and 4 × 10^4^ PM-1 T cells for 7 days in a 95% humidified incubator with 5% CO_2_ at 37°C.

### Detection of p24.

p24 content in culture supernatant was measured using an enzyme-linked immunosorbent assay (ELISA) (AALTO, IRE) or by a high-sensitivity RETROTEK p24 ELISA kit (Gentaur), where lower levels of p24 were produced.

### Statistical analysis.

Graphs show mean values with standard deviation error bars. One-way analysis of variance (ANOVA) followed by unpaired *t* test with Dunnett's correction was used to compare the different antibodies with the CH65 isotype control antibodies. All statistical analyses were performed using Prism 7 (GraphPad Software, Inc., La Jolla, CA).
